# Flow Preserving Endovascular Treatment of Traumatic Pseudoaneurysms of the Distal Anterior Cerebral Artery—Case Reports and Review of Literature

**DOI:** 10.3390/brainsci12050634

**Published:** 2022-05-11

**Authors:** Petr Krůpa, Antonín Krajina, Miroslav Lojík, Jaroslav Adamkov, Tomas Česák

**Affiliations:** 1 Department of Neurosurgery, Faculty Hospital, Faculty of Medicine in Hradec Kralove, Charles University, 50346 Hradec Kralove, Czech Republic; jaroslav.adamkov@fnhk.cz (J.A.); tomas.cesak@fnhk.cz (T.Č.); 2 Department of Neuroregeneration, Institute of Experimental Medicine, Czech Academy of Sciences, 14220 Prague, Czech Republic; 3 Department of Radiology, Faculty Hospital, Faculty of Medicine in Hradec Kralove, Charles University, 50346 Hradec Kralove, Czech Republic; antonin.krajina@fnhk.cz (A.K.); miroslav.lojik@fnhk.cz (M.L.)

**Keywords:** distal anterior cerebral artery, traumatic pseudoaneurysm, stent-assisted coiling

## Abstract

Traumatic intracranial pseudoaneurysms (tIPAs) are a very rare pathology caused by blunt or penetrating head trauma. Diagnostic and therapeutic challenges of tIPAs are due to their unpredictable onset during the initial injury, or in a delayed manner, their unclear traumatic mechanism. Moreover, the presence of subarachnoid, subdural, or intraventricular hematoma may often cause them to be overlooked, which can potentially be followed by lethal rebleeding. Treatment of these lesions is controversial and on a case-by-case basis with regard to endovascular therapy or open surgery. We report two cases of three tIPAs of the distal anterior cerebral artery (dACA) with immediate and delayed onset after the trauma. Endovascular therapy resulted in complete obliteration of lesions with flow preservation in the parent artery using the flow diverter-assisted coiling strategy. The aim of this manuscript is to discuss the mechanism, angioanatomical characteristics, and current treatment options for these exceptional lesions.

## 1. Introduction

Intracranial pseudoaneurysms (IPA) are a rare nosological entity representing less than 1% of all intracranial aneurysms. More importantly, they cause a total morbidity of 20% [1]. The most common cause of IPA is direct or indirect head trauma causing a blunt or penetrating head injury [2]. Whereas non-traumatic aneurysms are more common in middle-aged adults, traumatic intracranial pseudoaneurysms (tIPA) have higher incidence in children and young adults [3,4,5]. The most common locations of tIPAs are the middle cerebral artery (MCA), the supraclinoid segment of the intracranial carotid artery (ICA), the posterior cerebral artery (PCA), or their branches, and the distal anterior cerebral artery (ACA). Traumatic distal ACA (dACA) aneurysms have been reported to represent 30–40% of tIPA cases [6,7]. The mechanism of development of tIPAs of dACA following blunt trauma is most probably by the impact of the mobile arterial wall against the rigid dural duplicator—falx cerebri [4,8]. Clinical presentation of tIPAs is associated with headaches, intracranial hemorrhage, epistaxis, seizures, and neurological deficits. Patients with no evidence of vascular injury during the initial examination or primary surgical exposure may suffer postoperative or delayed hemorrhage.

Presence of a tIPA should be considered in any atypical intracranial hematoma in the near proximity of the larger feeding artery. Early detection using the CT angiography (CTA) with subsequently performed digital subtraction angiography (DSA) is mandatory.

The treatment options for tIPAs are either conservative management or an active approach via microsurgical or endovascular techniques. Neurosurgical techniques include clipping, wrapping, trapping, or resection of the pseudoaneurysm with a reconstruction of the flow. Although the direct surgical approach is risky due to the high rate of intraoperative ruptures, it can effectively decompress the surrounding hematoma and thus lower the intracranial hypertension [9]. Endovascular therapy has in its armamentarium coiling, stent-assisted coiling, flow-diverter implantation, or occlusion of the parent artery. Due to the fragility of the pseudoaneurysm, endovascular treatment is delicate and requires an experienced team.

The aim of this manuscript is to present treatment options in rare cases of tIPAs of dACA following penetrating and blunt trauma accidents treated in our department. Currently, there are no guidelines or larger prospective studies on treatment of tIPAs. We are presenting two case reports that were treated by endovascular techniques focused on preserving the flow in the parental artery.

## 2. Case Report

### 2.1. Case Report 1

**Figure 1:**
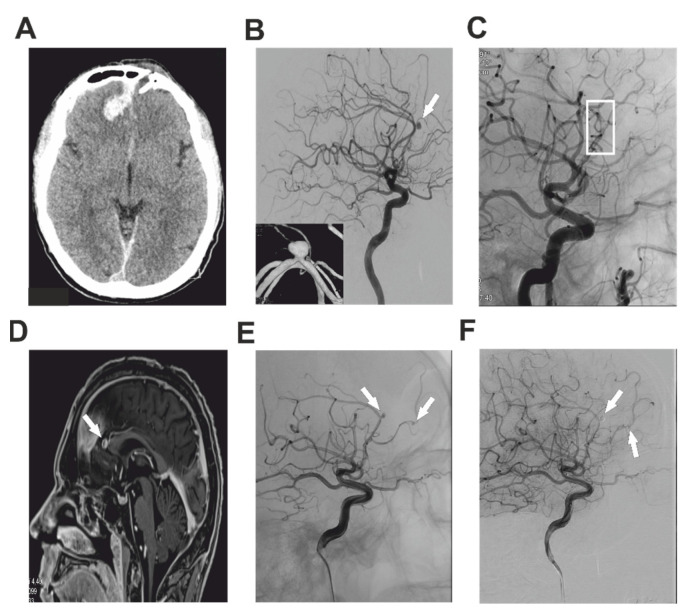
Imaging studies describing Case 1. Post-traumatic preoperative CT of the head showing intracerebral hematoma of the right frontal lobe with bone defect of the left side (**A**). DSA investigation with the white arrow pointing at the tIPA of the callosomarginal artery (**B**). DSA after treatment of the pseudoaneurysm using stent implantation (white box) (**C**). White arrow pointing on the growing remnant of the aneurysm of dACA several weeks after the primary treatment (**D**). Repeated DSA investigation. White arrowsshowing the growing partially-treated callosomarginal tIPA together with newly formed tIPA of the frontopolar artery on the right side (**E**). After successful embolization of both tIPAs, control DSA showed no residual flush in the tIPAs (white arrows) (**F**).

A 37-year-old healthy male was presented to the senior consultant of the Department of Neurosurgery, University Hospital Hradec Kralove, after having fallen onto an iron bar during a state of alcohol intoxication. An acutely performed CT of the head showed a penetrating open injury of the frontal lobe through the left frontal sinus accompanied by a minor intracerebral hematoma and interhemispheric subdural hematoma (Figure 1A). CTA showed no pathology of the intracranial arteries. An urgent neurosurgical revision was indicated with debridement of the wound and dural reconstruction. The postoperative course of the patient was without complication and he was dismissed on the 8th postoperative day. Six months after the trauma, the patient suffered his first seizure attack. CT and subsequent CTA of the brain were pursued and showed two small parafalx hemorrhages and a tiny tIPA of the callosomarginal artery on the right side. The patient was admitted to our department for a thorough DSA investigation, which proved a tIPA of size 4 × 3 mm of the callosomarginal artery (Figure 1B). The parent artery was also stenotic to 50% before and after the tIPA. DSA findings together with possible treatment options and perioperative risks were discussed with the patient and the endovascular treatment was chosen as a first-line therapy. An attempt at coil embolization was made, but the operation was complicated by temporary thrombosis of the A2-3 segment of the ACA. The thrombus was successfully dissolved using an infusion of Integrillin and the neck of the tIPA was treated by the implantation of an Atlas stent (3 × 15 mm) (Stryker Neurovascular, Cork, Ireland) (Figure 1C). No new neurodeficit was observed after the operation. However, a 2 mm remnant neck of the tIPA was observed on the postoperative MRA. A watch and wait strategy was chosen mainly due to the perioperative thrombosis. The patient was dismissed on the third postoperative day and control MRA was scheduled. After several weeks, the control MRA showed enlargement of the neck of the tIPA (Figure 1D) and endovascular treatment was urged. During repeated endovascular angiography not only was found the partially secured tIPA of the callosomarginal, but also a newly-formed tIPA of the frontopolar artery on the right side (Figure 1E). Firstly, the frontopolar artery was probed using an SL-10 microcatheter, and using guidewires Synchro-14 (Stryker Neurovascular, Cork, Ireland), Hybrid-8 (Balt Extrusion, Montmorency, France), and Synchro-10 (Stryker Neurovascular, Cork, Ireland) an Atlas stent (3 × 15 mm) was implanted (Stryker Neurovascular, Cork, Ireland), with subsequent embolization of the tIPA with 1.5 mm and 1 mm Nano coils. Secondly, the pericallosal artery was probed and using exchange guidewire Transend Floppy (Stryker Neurovascular, Cork, Ireland) and Headway-21 (MicroVention Europe, Saint Germain-en-Laye, France) the tIPA was covered with a FRED flow-diverter (MicroVention Europe, Saint Germain-en-Laye, France) 3 × 13 mm with subsequent embolization of the tIPA. The operation was performed under full dual antiaggregation using acetylsalicylic acid and clopidogrel and administration of 6000 units of heparin. Total obliteration of both tIPAs was achieved (Figure 1F). After the operation, the patient was without any neurodeficit and was discharged on the third postoperative day. After 3 months, a control MRA was performed and showed no filling of either tIPA. The patient will be regularly followed up.

### 2.2. Case Report 2

**Figure 2:**
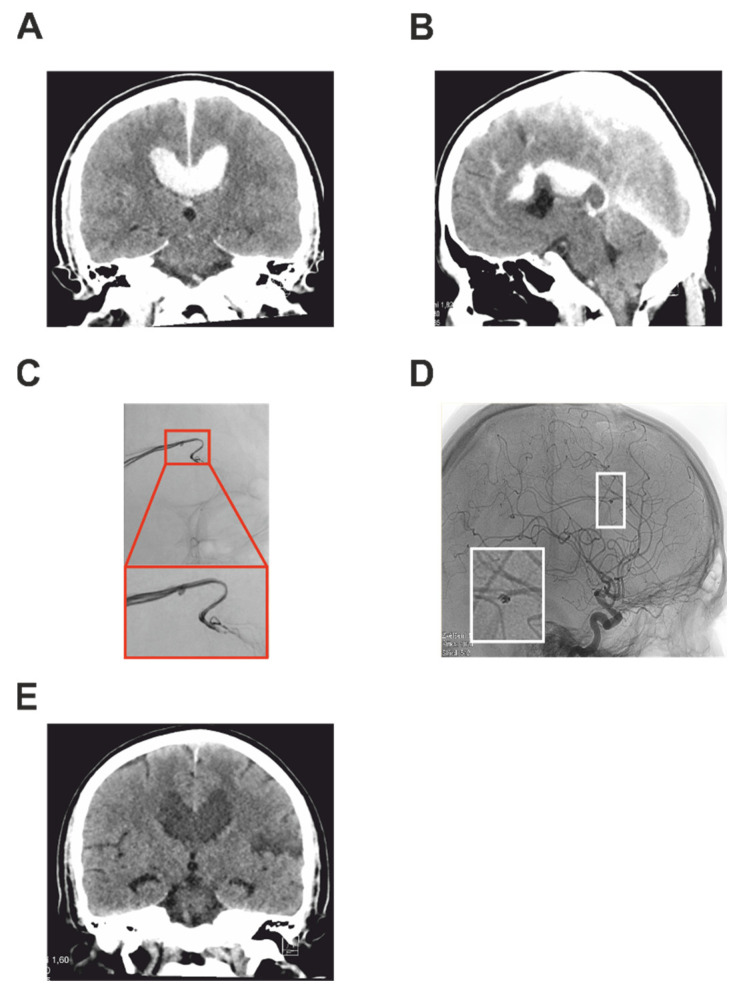
Imaging studies describing Case 2. Post-traumatic preoperative CT of the head showing intracerebral pericallosal hematoma in coronal (**A**) and sagittal section (**B**). DSA investigation of the tIPA of the pericallosal artery together with the detailed view in the red box (**C**). DSA after the coiling of the pseudoaneurysm with a detailed view in the white box showing the coils (**D**). Control coronal CT showing the hematoma one month after the trauma (**E**).

A 42-year-old man was presented after a serious motorcycle accident. Immediately after the accident, he was conscious, with serious fractures to his left lower limb. He was sedated and intubated and transported by air rescue service into the trauma center of University Hospital Hradec Kralove. A trauma protocol whole-body CT and CTA were performed, showing multiple extremity bone fractures and also an average size pericallosal hematoma deforming the corpus callosum (Figure 2A,B). The finding on CTA was suspicious of a tIPA on the pericallosal artery (AP) on the left. The patient was admitted for acute treatment for hemorrhagic shock and acute fractures. After stabilization of the patient, a control CT of the head was performed and showed a mild progression of the pericallosal hematoma. A subsequently performed brain DSA confirmed a tIPA (size 1.3 mm) of the AP amenable to endovascular treatment (Figure 2C). A microsurgical approach was discussed mainly due to the pericalosal hematoma, however, endovascular approach was chosen as a first-line therapy as a less invasive method in the polytraumatized patient. The patient was secured and transported to the endovascular operating room. Using the switch guidewire 260 Bentson (Cook Medical, Bloomington, IN, USA), a 6F guidewire was inserted and the AP was probed by an Infinity 260 Bentson microcatheter (Cook Medical, Bloomington, IN, USA). A slow infusion of 1 mL Dilceren was administered for the prevention of vasospasm. In the second step, new guidewires SL-10 and Synchro-14 were inserted to the near proximity of the tIPA and a Nano coil 1 × 30 mm was applied. Normal flow in the AP was checked 10 min after the coiling (Figure 2D). After the operation, the patient was admitted to the surgical intensive care unit (ICU) and next day extubated. During the neurocheck, a new severe right-sided hemiparesis and mild expressive aphasia were noted. A control brain CT showed a new, well-demarcated ischemia of the pericentral area on the left. There was no remnant of the tIPA and there was a patent flow of PA on the CT angiography. There was no enlargement of the pericallosal hematoma, which followed natural on the control CT (Figure 2E). After several reconstructive surgeries for bone fractures and a long rehabilitation, the patient recovered well and the hemiparesis regressed to only a mild level. Six months after the trauma, control imaging was performed with no signs of recurrence of the tIPA.

## 3. Discussion

Traumatic pseudoaneurysms of the distal anterior cerebral artery are a rare complication following penetrating or blunt head trauma. Several mechanisms have been proposed to explain the formation of a tIPA depending on the initial force. Direct penetrating trauma causes tIPAs, usually by bone fragments, various missiles, or sharp stabbing weapons. These may undoubtedly lead to injury of the arterial wall with the formation of the pseudoaneurysm. High-energy missiles tend to completely disrupt the continuity of the vessels, whereas lower-velocity bullets and their fragments tend to injure and tear the vessel wall without transecting the lumen. Therefore, low-energy missiles have a higher probability of developing a tIPA. On the other hand, blunt head injury leads to indirect vascular trauma by impact of the vessel wall against a bony or dural spur—the falx, the tentorium, or the sphenoid ridge [1]. Most of the tIPAs on the proximal cerebral arterial trunks are caused by penetrating trauma, whereas 60–70% of tIPAs on peripheral branches are caused by blunt injury. From a histological point of view, traumatic arterial aneurysms can be classified as true or false. True aneurysms develop as a result of a partial disruption of the arterial wall. The intima, internal elastic lamina, and media are damaged, whereas the adventitia is intact. False aneurysms (pseudoaneurysms) develop from disruption of the entire arterial wall. A hematoma forms outside the vessel and is restricted by perivascular connective tissues. However, it still communicates with the lumen of the injured artery and is more likely to rebleed. Regardless of the precise mechanism of formation of a tIPA of the dACA, due to their dissection origin, such aneurysms are highly unstable, with a high rate of rebleeding, and have to be considered as complex aneurysms requiring brisk therapy.

Following severe traumatic brain injury, imaging studies are mandatory. Computed tomography (CT) remains the gold standard for first-line imaging and is often supplemented with CT angiography (CTA). In the event that CTA confirms tIPA, the patient is transported as soon as possible to the digital subtraction angiography (DSA) workstation. The typical finding of the DSA is a globular aneurysmal sac without a neck, with late filling and stagnation of the contrast agent. In the case of a negative finding on DSA, repeated examination is considered in the following days to rule out delayed tIPA. The optimal timing for repeated DSA is still a matter of debate. Magnetic resonance angiography (MRA) is not usually employed in the acute phase as it has much lower sensitivity in tIPAs [10].

Treatment of dACA tIPAs is complex because they pose a real challenge due to the combination of a small parent vessel with limited collateral circulation. The lack of a neck and a well-formed wall make these pseudoaneurysms difficult to treat either by microsurgical or endovascular techniques and require case-by-case decision making.

In the past, most dACA tIPAs were treated surgically. Simple clipping of the pseudoaneurysm is often not feasible due to the absence of the aneurysmal neck and a proper wall. Thus flow remodeling by trapping with various revascularization strategies requiring EC-IC or IC-IC bypass may be chosen. With regard to the EC-IC bypass, it is usually quite a complicated procedure as the superior temporal artery (STA) is rarely long enough to reach the interhemispheric fissure. Revascularization, therefore, requires the harvesting of the arterial graft with two microanastomoses which increase the rate of bypass failure. However, the bypass occlusion rate in high-flow centers is probably low—Lawton et al. published a 97% patency rate in their exceptional series of 430 bypasses [11]. The IC-IC bypass strategy mostly involves two techniques—revascularization of the sacrificed branch via reimplantation on a branch originating from the contralateral A2 via end-to-side anastomosis (“after” aneurysmal trapping or excision), or revascularization of the sacrificed branch via side-to-side anastomosis (in the pericallosal cistern) with a branch originating from the contralateral A2 (“before” aneurysm trapping) [12]. However, IC-IC bypass procedures are “bilateral revascularization” surgeries and carry a significant risk of anastomosis occlusion with subsequent bilateral ischemic symptoms. Notably, the microsurgical procedure is still challenging due to the infrequent surgical field and a greater possibility of premature rupture causing complications during surgical dissection in the acute and is associated with significant morbidity and a reported mortality of 18–29% [13,14]. 

Non-surgical treatment includes various types of endovascular therapy for tIPA. The distal location makes endovascular access sometimes difficult (especially for techniques aiming at parent artery preservation). The deconstructive treatment of a tIPA by occluding the injured artery requires sufficient collateral flow [15]. Grossberg et al. reported that ACA occlusions may cause hemiparesis, motor neglect, apraxia, abulia, aphasia, and mutism due to the involvement of the supplementary motor area, corpus callosum, anterior diencephalon, and deep neural structures [16]. According to Park et al., most often is presented motor deficit on the contralateral lower limb with a favorable long-term outcomes [17]. On the other hand, Moshayedi et al. showed no significant neurological decline after the occlusion test for fusiform aneurysm of dACA enabling safe PAO with a good recovery [18]. Reconstructive treatment modalities involve closing the site of the injury with coils while preserving the parent artery, sometimes utilizing remodeling tools such as a balloon, a stent, or a flow diverter. The use of a stent or a flow diverter requires long-term use of anti-platelet treatment to prevent in-stent thrombosis. Cagnazzo et al. reported 19% symptomatic ischemic complications in their series of dACA aneurysms treated by flow-diverter stents [19]. In addition, reconstructive treatment is associated with a higher risk of recanalization of the injured site as well as a higher risk of the recurrence of bleeding, when coils can sit into the perivascular hematoma, and pulsation of the parent artery may push them out, with subsequent rebleeding [20,21].

In this work, we have presented two cases of newly developed tIPAs in young adults with early and delayed onset following penetrating and blunt head injury. 

Case 1 presented a delayed formation of multiple tIPAs following the direct trauma of the vessel wall. Despite suspicion of the arterial trauma during the initial trauma due to the proximity of the penetrating object, no such signs were observed on the CTA nor during the surgical revision. However, as in our case, it is known that even angiography is often negative and 20% of traumatic aneurysms may regress, enlarge, or form late [22,23]. To the best of our knowledge, this is the first case describe with delayed onset of multiple tIPAs following the direct trauma. A neurosurgical revascularizaton strategy was discussed, but such previous surgical approaches have increased the risk of fibrotic changes around the artery with higher peroperative morbidity risk. An endovascular approach was therefore chosen as a safer and comparable method of the treatment. Endovascular treatment of tIPAs often carries a risk of premature rupture of the fragile tIPA’s wall. This case was complicated by a temporary thrombosis of the parent artery, and hence the definitive treatment of the tIPA was two-staged with stent implantation and subsequent coiling. During the second endovascular session, a newly developed second tIPA was found and treated as well. Yuen et al. reported a case with recurrence of tIPA after coil embolization, and 6-month follow-up angiography showed aneurysm regrowth with migration of the coils. In this case, there was rapid enlargement of the aneurysm, as observed several weeks after the first embolization. However, a small neck was left intentionally, so a close follow-up was pursued [24].

Treatment decision-making in Case 2 was affected by the presence of a traumatic intracerebral hematoma, which was compressing the corpus callosum. Surgical management has the advantage of rapid decompression of the involved structures. However, it is well known that direct surgery for traumatic dACA aneurysm has a higher risk (18–22%) of surgical complications than other supratentorial aneurysms [1]. The main reason was the missing of the aneurysm neck, a thin aneurysm wall, and the high risk of re-rupture. Notably, the interhemispheric approach, which is most commonly used for pericallosal artery aneurysms has a limited surgical field due to the presence of bridging veins [14].

Most of the previous described cases of tIPA of the pericallosal arteries were treated by parent artery occlusion (PAO) [14,25,26,27]. It has been reported that neurologic deficits or new infarct areas are not often observed in distal territories if PAO was performed in the short segment, most likely because of the leptomeningeal collateral supply [26]. By using n-butyl cyanoacrylate glue, aneurysms and parent vessels can be effectively occluded and recurrence due to retrograde collateral circulation can be prevented. Hence PAO can be a safe and appropriate treatment option [27]. In our cases, we used a flow preserving method by stent-assisted coiling of the pseudoaneurysm leaving the parent artery patent. However, even if the flow was preserved, as it was in our case, there may be a new neurological deficit secondary to the cortical ischemia caused by temporal arterial spasm or microembolization. Despite the moderate neurological deficit, which was secondary to the endovascular treatment, gradual recovery of the neurological functions was achieved, and six months after the therapy, no major deficit was observed.

## 4. Conclusions

Endovascular treatment by stent-assisted embolization of traumatic pseudoaneurysms of the distal anterior cerebral artery has been shown as a viable and safe option for achieving elimination of the pseudoaneurysm while preserving flow in the parent artery.

## Data Availability

The data that support the findings of this study are available on request from the corresponding author, Petr Krůpa. The data are not publicly available due to containing information that could compromise the privacy of research participants.
